# Domestic tethers: Gender differences in career paths and domestic responsibilities of top-research medical school graduates

**DOI:** 10.1371/journal.pone.0267288

**Published:** 2022-04-20

**Authors:** Eveline Hitti, Dima Hadid, Samia J. Khoury, Hani Tamim, Maha Makki, Charlotte M. Karam

**Affiliations:** 1 Department of Emergency Medicine, American University of Beirut Medical Center, Beirut, Lebanon; 2 Abu Haidar Neuroscience Institute, American University of Beirut, Beirut, Lebanon; 3 Department of Internal Medicine, American University of Beirut, Beirut, Lebanon; 4 Telfer School of Management, University of Ottawa and Olayan School of Business, American University of Beirut, Beirut, Lebanon; MD Anderson Center, UNITED STATES

## Abstract

**Introduction:**

Gendered differences in career paths of medical graduates persist globally. We aim to explore the impact of domestic tethers on the career paths of physicians by studying gendered differences in domestic burdens of physicians as well as differences in perceptions around the impact of domestic work on professional advancement.

**Methods:**

A web-based survey including 38 questions was sent to all 3866 physician alumni of the top academic medical school in Lebanon. Data was collected between November 2018 and January 2019, with up to three invite reminders. Overall, 382 were included in the final analysis, 124 women (32%), 258 men (68%).

**Results:**

The study had a response rate of 10.4%. Findings show that a greater percentage of men were married and had children (77.5% vs 62.1%, p = 0.004, 77.9% vs 51.6%, <0.001, respectively). Majority of both women and men held full-time positions (82.1% and 87.1%), having children however reduced the odds significantly [OR = 0.2, 95% CI: (0.1–0.6), p = 0.01]for women, while only older age reduced it for men (OR = 0.1,95% CI: (0.04–0.2), p<0.001]. Among full-time physicians, men and women spent similar time on professional activities (60.2hrs/wk vs 58.3hrs/wk, p = 0.32). Women spent more time on parenting and household work (23.5hrs/wk vs 10.4hrs/wk, <0.001; 8.9hrs/wk vs 6.0hrs/wk, p = 0.001, respectively). Women physicians’ spouses contributed to 14.5 hours/week of total time on domestic activities whereas men physicians’ spouses spent two folds more time on domestic activities (35.0 hours/week, P<0.001). Of physicians with children, a higher percentage of women than men reported that children prevented their career advancement or their participation in development opportunities (43.8% vs 15.9%, p<0.001; 50.0% vs 19.4%, p<0.001, respectively). A greater percentage of women than men scaled back their career after first child (31.3% vs 3.5%, <0.001). Of married/partnered physicians, fewer women than men reported their career took priority over their partner’s when conflicts arose, (52.0% vs 86.0%, p<0.001).

**Conclusion:**

These findings highlight the heavier impact of domestic tethers on the career paths of women physicians than men physicians. Men are more likely than women to hold full-time positions in the early advancement defining phases of their careers. Full-time women shoulder more domestic work than men and experience more professional advancement concessions. Closing persistent gender gaps in medicine requires addressing inequities in domestic burdens through strategies that include mentorship on domestic tethers, support of on-site child-care and advocacy for parental leave policies that encourage shared care-work.

## Introduction

The gender gap at the medical training level has closed in many countries. In the US, women now make up half of incoming medical students. In multiple European countries, the number of woman physicians in training has exceeded 50% [1, 2]. This trend has also emerged in low-middle income countries including Lebanon where, over the past decade, women medical student enrollment has increased to 47% [3–5]. Despite much effort to equalize entry to medical schools, gender differences in career paths have persisted with more women choosing part-time career paths and fewer woman advancing to leadership positions in both academic medicine and across health care systems in general.

Literature on persistent gender disparities in the profession has focused on structural, organizational, and personal barriers to woman advancement [6–8]. Institutions over the years have responded by offering flexible working hours, extension of probationary periods for promotion reviews [9], implicit bias training, and improving mentorship programs [10]. Despite these efforts, and now decades after closing the medical school pipeline gap in some countries, recent data shows that women continue to represent a lower percentage of the workforce [11], with many dropping to part-time work or leaving the profession entirely [12]. While part-time schedules may help retain women in the workforce, especially during periods when parenting demands peak, this “off-tracking” can hurt career advancement and promotion, in contrast to men who usually experience fewer interruptions of full-time paths throughout their careers [8, 13].

While recent studies have started to assess the impact of parenting and household work on women’s decision to follow non-fulltime paths, few studies have looked at gender differences in domestic responsibilities and how household/parenting burdens impact attrition and advancement of men and women differently. This concept of “domestic tethers” places the inequities in domestic burdens at the core of persistent gender inequities within the profession.

This study explores the concept of domestic tethers within the medical profession by assessing gender differences in career paths of medical graduates from the top-research medical school in Lebanon. We aim to explore variables that contribute to part-time career paths. Furthermore, we aim to assess gender differences in time spent on professional and domestic work as well as perceptions around impact of domestic/parenting work on career advancement.

## Methods

### Study setting and design

A web-based survey of 38 questions was designed comprising six domains to assess demographic, family life, professional career paths, time spent on professional and domestic work, and perceptions around impact of domestic work on career advancement. Domestic work, herein, refers to combination of both parenting and household work. The survey was administered to physician graduates of the Faculty of Medicine (FM) at the American University of Beirut (AUB) whose email contacts were available through the alumni office. At the time of the study, the total list included 3,866 alumni, 34% of whom are women. Data collection was carried out between November 2018 and January 2019. Electronic informed consent was emailed with the survey. Three calls for participation were sent out. Ethical approval was obtained from the Institutional Review Board (IRB) of the American University of Beirut under the protocol number [SBS-2018-0415].

### Measurements

The authors based the questionnaire on published peer-reviewed literature that examines work-home conflict physicians’ experience [14, 15]. To enhance content validity, the questionnaire was customized to the institutional setting and reviewed by a group of experts, including a statistician, the director of the Emergency Medicine Department, the director of the Center of Inclusive Business and Leadership for Women and a social scientist. The expert team modified the questionnaire as necessary, and a consensus was reached on the final draft, which was then piloted on 20 medical graduates. All feedback was consolidated in the final version of the survey. Prior completing the questionnaire, an electronic consent form needed to be signed.

Demographic questions included age, gender, marital status, employment status of the participants and their spouse, spouse’s current profession, number of children and the age at which the first child was born. Questions related to career included practice characteristics, specialty, current academic rank, years in practice, hours worked per week, number of nights on call per week. Specialties were categorized according to the Accreditation Council for Graduate Medical Education (ACGME) [16]. Men dominated specialties included: neurology, ophthalmology, otolaryngology-head neck surgery, radiology, emergency medicine, surgery, urology, anesthesiology, internal medicine. Women dominated specialties included: Obstetrics and gynecology, pediatrics, psychiatry, pathology, dermatology, family medicine. Time spent on domestic work was broken down into parenting and household work. Time spent at work included patient care, teaching, research and administrative activities. Attitude towards domestic burden and impact on career advancement included questions related to whether commitment to raising children slowed their career advancement or prevented career development, and how work-life conflicts were resolved.

### Statistical analysis

Data analysis was limited to non-retired participants. Descriptive analysis of the results was performed using IBM SPSS statistical software for Windows version 24 (SPSS for Windows, version 24; SPSS, Inc, Chicago, IL). Data were described as number and percent for categorical variables, whereas the mean and standard deviation (±SD) were calculated for continuous ones. In the bivariate analysis, the association between the employment status (full time/part time) among men and women and other categorical variables was assessed using Chi-Square test, whereas the Student’s t-test was used for the association with continuous variables. Moreover, multivariate stepwise logistic regression was carried out to identify the predictors of the outcome. In addition, stratified analysis based on gender was carried out to explore potential modifiers to gender and career paths. Results are presented as odds ratios (OR) and their corresponding 95% confidence intervals (CI). P-value < 0.05 was used to indicate statistical significance. Missing values were excluded by line.

## Results

There were 403 responders to the survey for a response rate of 10.4%. After excluding retired participants, a total of 382 participants were included in final analysis, 32% of whom were women. Demographic and professional characteristics of the participants are summarized in **Table 1**. Surveyed women physicians were relatively younger than their men counterparts (mean age 42.4±13.0 vs 51.4±15.9). A greater percentage of the men physicians were married (77.5% vs 62.1%, p = 0.004). A greater percentage of woman physicians however were married to spouses who were employed and had spouses with full-time employment (86.3% vs 45.5%, <0.001; 85.5% vs 58.7%, <0.001, respectively). Surveyed men tended to be in historically men dominated specialties (64.3% vs 43.5%, p<0.001). The majority of both women and men worked full-time and practiced in an academic setting (82.1% vs 87.1% and 62.4% vs 56.7% respectively).

**Table 1:** Participant personal and professional characteristics, by gender.

		Gender		
Demographic		Women	Men	p-value
		N = 124	N = 258	
**Age**	Mean (SD)	42.4±13.0	51.4±15.9	<0.001
	<36	44 (35.5)	44 (17.1)	<0.001
	36–50	50 (40.3)	79 (30.6)	
	51–65	23 (18.5)	87 (33.7)	
	≥66	7 (5.6)	48 (18.6)	
**Marital status**	Married/domestic partnership	77 (62.1)	200 (77.5)	0.004
	Single (never married)	37 (29.8)	50 (19.4)	
	Divorced or widowed	10 (8.1)	8 (3.1)	
	Married with kids	56 (72.7)	193 (96.5)	<0.001
	Married without kids	21 (27.3)	7 (3.5)	
**Children**	Yes	64 (51.6)	201 (77.9)	<0.001
**Number of Children**	1	10 (15.6)	17 (8.5)	0.01
	2	34 (53.1)	76 (37.8)	
	≥3	20 (31.3)	108 (53.7)	
**Age when had first child, (quartile)**	≤29	25 (39.1)	60 (29.9)	0.06
	30–31	18 (28.1)	43 (21.4)	
	32–34	13 (20.3)	40 (19.9)	
	≥35	8 (12.5)	58 (28.9)	
**Professional**				
**Contract type**	Full time	96 (82.1)	203 (87.1)	0.20
	Part time	21 (17.9)	30 (12.9)	
**Specialty**	Women dominated specialties^	60 (48.4)	77 (29.8)	0.001
	Men dominated specialties&	54 (43.5)	166 (64.3)	
	Others	10 (8.1)	15 (5.8)	
**Nights on call per week**	Yes	56 (52.3)	152 (68.8)	0.004
**Primary practice setting**	Private practice	30 (25.6)	77 (33.0)	0.36
	Academic medical center	73 (62.4)	132 (56.7)	
	Other	14 (12.0)	24 (10.3)	
**Current academic rank**	Instructor	2 (2.8)	4 (3.0)	0.13
	Assistant professor	13 (18.1)	36 (27.3)	
	Associate professor	15 (20.8)	27 (20.5)	
	Full professor	12 (16.7)	32 (24.2)	
	Other	30 (41.7)	33 (25.0)	
**Spouse**				
**Spouse employed**	Yes	69 (86.3)	92 (45.5)	<0.001
**Spouse’s contract type**	Full-time	59 (85.5)	54 (58.7)	<0.001
	Part time	10 (14.5)	38 (41.3)	
**Spouse’s current profession**	Medical doctor	35 (50.7)	25 (27.2)	<0.001
	Other health care professional	2 (2.9)	26 (28.3)	
	Non-medical professional	32 (46.4)	41 (44.6)	

^ Obstetrics and gynecology, pediatrics, psychiatry, pathology, dermatology, family medicine.

^&^ neurology, ophthalmology, otolaryngology-head neck surgery, radiology, emergency medicine, surgery, urology, anesthesiology, internal medicine.

### Gender differences among career paths

**Table 2** presents gender differences in demographics and career characteristics among full-time and part-time physicians. Among full-timers, the highest percentage of women was in the less than 36 years old age group (40.6%) whereas the highest percentage of men was in the 51–65 age group (37.4%). Among part-timers however, while the highest percentage of women was in the 36–50 age group (57.1%), the highest percentage of men was in the >66-year-old age group (46.7%). Within the full-time physician group, a higher percentage of men were married (74.4% vs 58.3%, <0.004), had children (72.9% vs 45.8%, <0.001), were in historically men dominated specialties (67.0% vs 52.1%, <0.03) and took night call (71.9% vs 58.6%, <0.03). For both full-time and part-time groups, women’s spouses tended to be employed compared to men physicians (91.4% vs 52.3%, <0.001; 88.2% vs 33.3%, <0.001, respectively) and have spouses with full-time jobs (90.6% vs 60.0%, <0.001; 66.7% vs 55.6%, p = 0.68).

**Table 2:** Gender differences in personal and professional characteristics among full-time and part-time physicians.

		Full-time			Part-time			Women	Men
		N = 299			N = 51				
Demographic		Women	Men	p-value	Women	Men	p-value	p-value (full-time vs part-time)	p-value (full-time vs part-time)
		N = 96	N = 203		N = 21	N = 30			
**Age**	**Mean (SD)**	40.7±11.8	46.7±12.7	<0.001	47.7±11.8	61.9±14.9	0.001	0.01	<0.001
	<36	39 (40.6)	43 (21.2)	<0.001	2 (9.5)	1 (3.3)	0.003	0.01	<0.001
	36–50	37 (38.5)	74 (36.5)		12 (57.1)	5 (16.7)			
	51–65	18 (18.8)	76 (37.4)		5 (23.8)	10 (33.3)			
	≥66	2 (2.1)	10 (4.9)		2 (9.5)	14 (46.7)			
**Marital status**	Married/domestic partnership	56 (58.3)	151 (74.4)	0.004	17 (81.0)	27 (90.0)	0.57	0.16	0.06
	Single (never married)	32 (33.3)	48 (23.6)		3 (14.3)	2 (6.7)			
	Divorced or widowed	8 (8.3)	4 (2.0)		1 (4.8)	1 (3.3)			
	Married with kids	37 (66.1)	144 (95.4)	<0.001	17 (100.0)	27 (100.0)	-	0.003	0.60
	Married without kids	19 (33.9)	7 (4.6)		-	-			
**Children**	Yes	44 (45.8)	148 (72.9)	<0.001	17 (81.0)	28 (93.3)	0.21	0.004	0.01
**Number of children**	1	9 (20.5)	14 (9.5)	0.01	0 (0.0)	1 (3.6)	0.09	0.03	0.01
	2	25 (56.8)	66 (44.6)		8 (47.1)	5 (17.9)			
	≥3	10 (22.7)	68 (45.9)		9 (52.9)	22 (78.6)			
**Specialty**	Women dominated specialties^^^	39 (40.6)	52 (25.6)	0.03	18 (85.7)	15 (50.0)	0.02	0.001	0.02
	Men dominated specialties^&^	50 (52.1)	136 (67.0)		3 (14.3)	15 (50.0)			
	Others	7 (7.3)	15 (7.4)		-	-			
**Nights on call per week**	Yes	51 (58.6)	138 (71.9)	0.03	5 (25.0)	14 (48.3)	0.14	0.01	0.01
**Primary practice setting**	Private practice	17 (17.7)	60 (29.6)	0.09	13 (61.9)	17 (56.7)	1.00	<0.001	0.004
	Academic medical center	67 (69.8)	123 (60.6)		6 (28.6)	9 (30.0)			
	Other	12 (12.5)	20 (9.9)		2 (9.5)	4 (13.3)			
**Spouse employed**	Yes	53 (91.4)	80 (52.3)	<0.001	15 (88.2)	9 (33.3)	0.001	0.65	0.07
**Spouse’s contract type**	Full-time	48 (90.6)	48 (60.0)	<0.001	10 (66.7)	5 (55.6)	0.68	0.03	1.00
	Part-time	5 (9.4)	32 (40.0)		5 (33.3)	4 (44.4)			

^ Obstetrics and gynecology, pediatrics, psychiatry, pathology, dermatology, family medicine.

^&^ neurology, ophthalmology, otolaryngology-head neck surgery, radiology, emergency medicine, surgery, urology, anesthesiology.

### Analysis of variables associated with part-time career

Multivariate logistic regression analysis was performed to identify predictors of part-time career paths (**Table 3**). Men had a 2.3 higher odds of having a full-time position than women (p = 0.02). Having children and older age reduced the odds of having a full-time position. Stratified analysis based on gender was carried out to explore potential modifiers to gender and career paths using multivariate logistic regression using the same variables (**Table 4**). For men, older age was the only variable associated with lower odds of full-time work (OR = 0.1, <0.001). For women, however, the only variables that increased the odds of full-time work was historically men-dominated specialty (OR = 6.8, <0.004), whereas having children reduced the odds significantly (OR = 0.2, p = 0.01).

**Table 3:** Multivariate logistic regression of predictors of part-time career paths (STEPWISE method).

Currently employed contract (Reference: part-time)									
	Chi-square	B	S.E.	Wald	df	P-value	OR	95% C.I.	
								Lower	Upper
Gender (men)	8.1	0.8	0.4	5.1	1	0.02	2.3	1.1	4.8
Age (≥65)		-2.4	0.5	27.3	1	<0.001	0.1	0.04	0.2
Specialty (men dominant specialty)		1.2	0.4	10.6	1	0.001	3.2	1.6	6.4
Children		-1.4	0.5	8.8	1	0.003	0.2	0.1	0.6

Imposed: gender (reference: Women).

Stepwise: Age (Reference: <36); marital status (Reference: single); specialty (Reference: Women dominated specialties); Children (reference: no).

**Table 4:** Multivariate logistic regression of potential modifiers of career paths, by gender (STEPWISE method).

Gender		Chi-square	B	S.E.	Wald	df	P-value	OR	95% C.I.	
									Lower	Upper
**Women**	**Specialty (men dominated specialties)**	0.8	1.9	0.7	8.1	1	0.004	6.8	1.8	25.3
	**Children**		-1.6	0.6	7.3	1	0.01	0.2	0.1	.6
**Men**	**Age (≥65)**	-	-2.8	0.5	33.4	1	<0.001	0.1	0.02	.2

Age (Reference: <36); marital status (Reference: single); specialty (Reference: Women dominated specialties); Children (reference: no).

### Gender differences in time allocation among full-time physicians

**Table 5** presents a comparison between the time allocated to household and parenting tasks by gender. Women physicians reported that on average they spent more time on domestic work than men physicians (19.9 hours/week vs 13.4 hours/week, p = 0.01). Their spouses however contribute to 14.5 hours/week of total time on domestic activities compared to men physicians whose spouses spend two folds more time on domestic activities (35.0 hours/week, P<0.001). This pattern was also apparent when household and parenting work were explored separately, with larger differences reported in parenting work where women physicians reported spending more than two-times the hours on parenting duties as compared to men physicians (23.5 hours/week vs 10.4hours/week, P<0.001 respectively). They also reported that their spouses contributed to an average of only 5.6 hours/week on household duties, while men physicians report that their wives provide nearly three-times that time (15.7 hours/week, P<0.001). As for the time allocated for work related activities, both men and women physicians indicated spending similar number of hours on professional activities including teaching, research and administrative service. The only significant difference was in the time spent on patient care activities, where men physicians spent 41.0 hours/week and women physicians 36.5 hours/week (P = 0.04).

**Table 5:** Gender differences in time allocation among full-time physicians.

			Full time		
			n = 299		
			Women	Men	p-value
			N = 96	N = 203	
**Time spent household and parenting tasks**	**Total domestic work (hours/week)**	Personal	19.9±21.3	13.4±15.4	0.01
		Spouse	14.5±15.7	35.0±26.4	<0.001
	**Household work (hours/week)**	Personal	8.9±7.6	6.0±5.0	0.001
		Spouse	5.6±5.0	15.7±9.7	<0.001
	**Parenting work (hours/week)**	Personal	23.5±20.7	10.4±16.0	<0.001
		Spouse	15.4±14.2	21.6±22.4	0.13
	**Professional work (hours/week)**	**Total**	58.3±15.2	60.2±14.6	0.32
		Time spent per week on patient care activities	36.5±16.5	41.0±15.8	0.04
		Time spent per week on teaching activities	7.5±5.2	8.0±6.1	0.53
		Time spent per week on research activities	14.5±15.4	11.7±16.2	0.26
		Time spent per week on administrative service activities	10.8±13.4	8.9±10.5	0.25

### Attitudes to domestic work and career advancement

**Table 6** describes the attitudes of physicians towards the impact of domestic work on careers and how work-life conflicts are resolved. Significantly more women (42.2%) indicated that they stayed home for child-illnesses or non-school days, as compared to the men who stayed home (3.0%, P<0.001). Of physicians with children, a higher percentage of women than men reported that children prevented their career advancement or their participation in development opportunities (43.8% vs 15.9%, p<0.001; 50.0% vs 19.4%, p<0.001, respectively). Finally, in terms of scaling back their careers after having the first child, 31.3% of women compared to 3.5% of men indicated that only they had to scale back their career but not their spouse (P<0.001). Moreover, when asked specifically about conflicts resulting from responsibilities at home and interfering with physicians’ career, more women perceived such conflicts to arise (92.2%) compared to men (86.0%). Furthermore, when asked about resolving the conflict, 52.1% of our sample of women physicians indicated that their career advancement took priority over their husbands compared to 86.0% of men (P<0.001).

**Table 6:** Work life-intersection and perception towards impact on career.

			Gender		
			**Women**	**Men**	**p-value**
			**N = 64**	**N = 201**	
**Children-related** ^*^	**Children prevented career advancement**	Yes	28 (43.8)	32 (15.9)	<0.001
	**Children prevented career development**	Yes	32 (50.0)	39 (19.4)	<0.001
	**Scale back the career after had first child**	Only me	20 (31.3)	7 (3.5)	<0.001
		Only my partner	2 (3.1)	88 (44.0)	
		Both	8 (12.5)	14 (7.0)	
		Neither	34 (53.1)	91 (45.5)	
	**Cared for child when ill/had non-school day**	I did/do	27 (42.2)	6 (3.0)	<0.001
		My spouse/partner	9 (14.1)	165 (82.1)	
		Another family member	7 (10.9)	4 (2.0)	
		Nanny	13 (20.3)	14 (7.0)	
		other	8 (12.5)	12 (6.0)	
			**Women**	**Men**	**p-value**
			**N = 77**	**N = 200**	
**Work-home conflicts** ^**&**^	**Experienced conflict between you and your spouse’s/partner’s career** ^**&**^	Yes	71 (92.2)	172 (86.0)	0.16
	**Whose career took priority the most recent time a conflict arose** ^**&**^	My spouse’s/partner’s career responsibilities took priority over mine	34 (47.9)	24 (14.0)	<0.001
		My career advancement took priority over that of my spouse/partner	37 (52.1)	148 (86.0)	

*Only asked of physicians indicating that they have children.

^&^Only asked of physicians indicating that they currently are married/partnered.

## Discussion

Gender gaps in the medical workforce and in leadership positions continue despite narrowing inequities at the level of medical education. This study explores gender differences in predictors of attrition from full-time career paths of physicians at the top research medical school in Lebanon, inequities in domestic work and the perceived impact of domestic work on career development and advancement. The majority of both women and men held full-time positions. Only having children, however, reduced the odds significantly of working full-time for women, whereas older age was the only variable that reduced the odds for men. Further exploration of gender differences in time spent on professional and domestic work revealed that, among full-time physicians, men and women spent similar time on professional activities. Women, however, reported spending more time on domestic work than men and their spouses contributed less time to domestic work than the spouses of the men physicians. Of physicians with children, a greater percentage of women than men reported that children prevented their career advancement or their participation in development opportunities and more women than men experienced work interruptions when home-work conflicts arose.

While some studies have started to explore gender differences of childcare on career paths of physicians and gender inequities in unpaid work amongst physicians, this study is the first to explore the interplay of inequity in unpaid work and career paths in a single cohort of physicians. A time-survey study of US physicians found that women physicians spent 100.2 min/day more on household and parenting activities than men physicians [17] but did not explore the impact on career paths of this group. Another study followed physicians prospectively and found that almost three quarters of women in the first 6 years post medical training reduced work to part-time or considered part-time work [18], with women more likely than men citing family reasons for their work status considerations. This study however did not explore the gender differences in time spent on domestic work within that cohort. Domestic tethers concept requires linking the two together, by exploring—at a granular level -inequities in household/parenting responsibilities and impact of these inequities on career progression and advancement.

The gender work-hour gap has been described in multiple contexts [19], with more women physicians leaving the practice or dropping down to part-time than men physicians, leading to diminishing percentage of women in full-time positions with time from graduation [18]. In the UK, within the National Health Service, the majority of women (63%) work part-time compared to only 8% of men. A study looking at physicians in Spain found that women graduates held more than twice the number of part-time positions as men graduates. Consequently, while men physicians were working on their first promotion, women physicians with similar years of experience had still not scaled up to full-time [20]. In our study, while the difference between women moving into part-time paths compared to men was small, it was clear that the move was occurring at early career for women and late career for men. Children and being in a women-dominated specialties emerged as the only variables associated with part-time path for women, and older age, for men. This supports the findings of Frank et al. in which women physicians who reported considering scaling back to part time, cited family reasons as a major factor in their decision [18]. This “off-tracking” of women early in their careers can explain the progressively widening gender gap with career progression and up the leadership ladder [21, 22].

Even though our study design did not allow for exploring gender differences in time spent on unpaid work prior to off-tracking, it is clear that differences exist in unpaid domestic work even for those physicians who continue to work full-time. Not only were women physicians spending more time on parenting and household work than their men peers, but they also had spouses who spent less time on these activities than the men physicians. The disproportionate burden was particularly prominent in parenting work, where women reported more than double the hours than their men peers. The larger difference in parenting work compared to household work could be explained by the latter (cleaning, cooking, home organizing) being more amenable to out-sourcing compared to parenting work. Disparities in household work between men and women have historically been rationalized economically, where men in the relationship are more likely to hold higher earning jobs. This, however, does not necessarily hold true for physicians where the earning potential of women in the relationship is high. In spite of this, repeat studies have found that inequities on the home-front exist for physicians, even within highly competitive specialties or cohorts early-career physician-researchers [6], academic faculty [23] and surgeons [24]. The existence of such disparities in repeat cohorts of highly competitive physicians suggests that the inequities go well beyond economic considerations, but rather lie in deeper rooted cultural/societal expectations where domestic work continues to be perceived as a woman’s primary responsibility. In addition to impacting paid-work effort, these heavy domestic burdens have been found to be associated with higher burn-out [24, 25] and lower career satisfaction [26], factors that can impact retention and advancement.

To understand whether these disparities impacted physician careers, or merely came at the expense of personal time, we asked physicians to reflect on whether “having children” affected their career in terms of advancement, development as well as pressure on them to scale back their careers. Significant gender differences in the responses were found, where women reported greater overall negative career impact, with more women than men reporting that having children: prevented their own career advancement, led to missed development opportunities, led to their scaling back their careers (not their spouse), and missing work during child illness/ non-school days. Furthermore, when we explored specific instances of work conflict due to domestic/ duties and the manner of resolution adopted, we found that significantly more women perceived such conflicts to arise than did men physicians. Furthermore, when asked about resolving the conflict, significant differences between whose career took priority in the resolution were clear, with only 57.7% of women indicating that that their career took priority over their spouses, compared to 82.7% of men physicians. Similar findings were also reported in a study of surgeons, a specialty on the higher end of the reimbursement scale and where workload, in general, is considered one of the most demanding [24].

These findings suggest that gender disparities in the burden of domestic work inevitably place women at a disadvantage compared to men. Not only are men at more liberty to invest additional time in work and remain in full-time positions in the early advancement defining phases of their career, but also experience work-life with fewer disruptions and more presence throughout their professional journey. In a qualitative study exploring the under-representation of women in leadership positions, one men respondent articulated this advantage as such: “I could at any time turn up to a meeting on a weeknight. I could be away overnight. I could do what I have to do to be noticed and available” [27]. Our findings are also consistent with other studies that have looked at development/advancement barriers and found that women are less likely to take sabbaticals than men and less willing to move to pursue advancement opportunities than men [28]. The incremental impact of such concessions could partly explain the widening gender gap along the leadership ladder.

The COVID-19 pandemic has led to significant disruptions in schooling, childcare and work-life boundaries. Recent studies have found that women physicians have been impacted by these changes to a greater extent than men physicians, with women reporting greater childcare/schooling responsibilities, taking on more work from home and experiencing greater work-life conflicts during the pandemic. Exploring these domestic tethers within the pandemic context and its long-term impact on the careers of women physicians is an area that needs close attention.

Addressing some of the institutional structural barriers to women in medicine have successfully narrowed the gender gap at the training level in many institutions. Without recognizing the persistent impact of gender inequities in domestic work on career sustainability and advancement, however, the pipeline will continue to leak at both the workforce level as well as the leadership level. Addressing these “domestic tethers” and the cultural expectations around childcare/household responsibilities are key to closing these persistent gaps. At the same time, institutions can play a substantive role to more centrally acknowledge carework and to build internal human resource policies and processes that facilitate a deeper understanding of the carework burden. Initiating training programs wherein healthcare administrators and educators are provided a meaningful understanding of the complexity of domestic responsibilities and the disproportionate level of burden shouldered by women is an important cornerstone for reimagining more equitable employment experiences in healthcare organizations. Our findings from current sample show that women’s carework marginalized them from participating fully and equally in paid work in healthcare settings. Proactive efforts by HR leaders could focus more on accounting for varying needs due to domestic tethers, and on providing women with pathways for retention and upward mobility opportunities, while simultaneously considering their wellbeing. Such efforts could include supporting on-site child-care, scheduling decision-making meetings and development opportunities to within regular work hours and supporting parental leave policies that encourage men as equal partners at home.

### Limitations

This study has some limitations. Firstly, given the survey methodology, response bias remains a possibility as do biases that come with self-reporting. No physicians who had left the workforce entirely responded. Given that this group is in general predominantly women, our conclusions related to domestic-professional associations are likely conservative. Secondly, the cross-sectional nature of the study hinders the ability to address causality. Finally, a greater proportion of women are in the younger age group which could skew the results related to domestic work experience.

## Conclusion

Men physicians are more likely than women to hold full-time positions in the early advancement defining phases of their careers. On the other hand, women report shouldering more domestic work than men. Women also experience more disruptions and professional advancement/development concessions throughout their career. Addressing inequities in domestic burdens is important to closing persistent gender gaps in medicine.

## Supporting information

S1 Appendix De-identified data set. (SAV)
